# Analysis of ticks (Acari: Ixodida) and associated microorganisms collected on the North Sea Island of Heligoland

**DOI:** 10.1007/s00436-025-08478-0

**Published:** 2025-03-17

**Authors:** Robert E. Rollins, Jochen Dierschke, Anna Obiegala, Heiner von Buttlar, Lidia Chitimia-Dobler, Miriam Liedvogel

**Affiliations:** 1 Institute of Avian Research “Vogelwarte Helgoland”, An der Vogelwarte 21, 26386 Wilhelmshaven, Germany; 2 Institute of Animal Hygiene and Veterinary Public Health, University of Leipzig, Leipzig, Germany; 3 Institute of Ecology and Environmental Sciences of Paris, Sorbonne Universitée, Campus Pierre et Marie Curie – Paris 5e, Tour 44-34, Paris, France; 4 Bundeswehr Institute of Microbiology, Munich, Germany; 5 Fraunhofer Institute of Immunology, Infection and Pandemic Research, Penzberg, Germany; 6 Experimental Parasitology, Department of Veterinary Sciences, Faculty of Veterinary Medicine, Ludwig-Maximilians-Universität, LMU, Munich, Germany

**Keywords:** Ixodida, *Haemaphysalis punctata*, *Ixodes ricinus*, Heligoland, Tick-borne pathogens

## Abstract

Heligoland is an island located in the North Sea, where vegetation was almost destroyed as a result of heavy bombardment during and after the Second World War. However, over the past 70 years, the vegetation has developed from scrub towards bushy or even forested environments. This change has most likely altered habitat suitability for various organisms, including many species of ticks. Ticks can act as major vectors for various pathogens of humans and animals; thus, characterizing the occurrence of a tick population and associated microorganism on the island is of great importance in relation to public and animal health. For this characterization on Heligoland, we flagged ticks at four different locations during June 2023 and 2024. In 2024, ticks were opportunistically sampled from house pets living on the island and during the annual ringing of common murre (*Uria aalge*) fledglings. In total, 267 ticks were collected over the 2 years which were identified morphologically, and confirmed molecularly if needed, to four species: *Ixodes ricinus* (*n* = 132), *Haemaphysalis punctata* (*n* = 47), *Ixodes uriae* (*n* = 3), and *Alectorobius maritimus* (*n* = 85), which for the latter represents the first report in Germany. Questing tick samples positive for *Borrelia burgdorferi* sensu lato, *Rickettsia* spp., *Anaplasma phagocytophilum*, and *Babesia* spp. were found in one or both years. Subsequent sequencing showed the presence of two *Rickettsia* species (*R. helvetica*, *R. aeschlimannii*), multiple *Borrelia* species (*B. garinii*, *B. valaisiana*, *B. bavariensis*, *B. afzelii*), and two *Babesia* species (*Ba. venatorum*, *Ba. capreoli*). Our research highlights a diverse tick and associated microorganism population on the island, which could pose public and animal health risks that will need to be monitored in the future.

**Supplementary Information:**

The online version contains supplementary material available at 10.1007/s00436-025-08478-0.

## Introduction

The island of Heligoland is located in the North Sea and consists of two sub-islands that are often frequented as a tourist destination (Dierschke et al. 2011). The island has a long and tumultuous history having sustained heavy bombing during the Second World War, and after being used as a bomb testing facility, which resulted in changes to the overall habitat of the island. This has resulted in the terrestrial ecosystems and associated vegetation present on the island having established over a relatively short time frame (< 100 years). The change in vegetation has led to different habitat types across the island with portions of the island now dominated by grassland, bushy habitat, and even small forests (Dierschke et al. 2011). This has made the island habitable for further organisms, such as species of ticks (Acari: Ixodida), which could pose potential risks to public health due to their role as vectors of various human and animal diseases (Estrada-Peña et al. 2017). Ticks are the most medically important vector of various diseases to animals worldwide and are only rivaled by mosquitos in their importance to vector-borne disease in humans (Estrada-Peña et al. 2017). Ticks have been shown to arrive with migratory birds using Heligoland as a stopover site (Walter et al. 1979), creating a possibility for introduction and establishment. Additionally, Heligoland hosts a large diversity of breeding seabirds which are also known to harbor specific tick species (Dietrich et al. 2010). The presence of one of these tick species, *Ixodes* (*I.*) *uriae*, was already reported as the only known location of this species in Germany (Rubel et al. 2021). Due to these factors, it is quite likely that local tick species do exist on the island and warrants further investigation.

Within this study, we aimed to (1) determine if a questing tick population does exist on Heligoland, and if so, which associated microorganisms are present, and (2) determine if seabird associated tick species, such as *I. uriae*, are still found in the seabird breeding colonies of the island.

## Methods

**Fig. 1:**
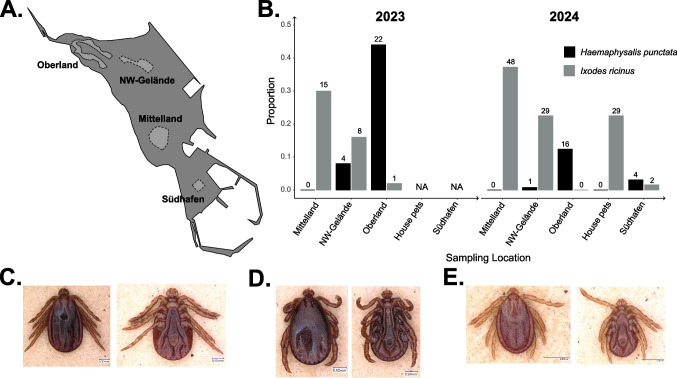
Overview of questing ticks collected on Heligoland from 2023 to 2024. **A** Schematic of the main island of Heligoland with tick sampling locations marked in light gray. **B** Proportion of all flagged questing ticks (see Table 1 for breakdown by tick life stage) in a given year per collection site. Color corresponds to tick species (*Ha. punctata*, black; *I. ricinus*, gray). The numbers above the bars refer to the absolute number of questing ticks collected at each site in a given year. **C** Pictures of a *Ha. punctata* adult, female flagged in the NW-Gelände collection site. **D** Pictures of an adult, male *Ha. punctata* collected from an employee of the Institute of Avian Research “Vogelwarte Helgoland” (not included in questing tick analysis as questing location on the island could not be determined). **E** Pictures of a *Ha. punctata* nymph flagged in the Oberland collection site. Images were taken using VHX900 Keyence microscope

Ticks were collected in 2023 (June 10–15) and 2024 (June 12–19) through drag sampling in four locations covering different habitat types of the island, locally referred to as Oberland (grassland; GPS: 54.1870° N, 7.8752° E), NE-Gelände (bushy habitat; GPS: 54.1861° N, 7.8827° E), Mittelland (small forest; GPS: 54.1795° N, 7.8851° E), and Südhafen (grassland, 54.1758° N, 7.8911° E); see Dierschke et al. (2011) for further information on the chosen areas (Fig. 1A). Südhafen was only sampled in 2024 while all other plots were sampled in both years. For sampling, a 1 × 1 m^2^ cotton cloth was dragged over vegetation for 10 m before being turned over and assessed for attached ticks. Sampling was performed each morning at one site for 4 h (8:00 AM–12:00 PM). Each location was sampled twice per year on non-consecutive days (i.e., 8 h in total). Additional, opportunistic sampling was undertaken in 2024, where ticks were collected, by owners, from house pets (cats, *n* = 2; dogs, *n* = 1) who live full time on the island. Furthermore, in 2024 during the annual ringing effort of the fledging common murre (*Uria aalge*) chicks, living individuals as well as naturally deceased chicks found at the ringing site were examined for attached ticks, which were removed with forceps if found. Due to the 2022–2023 H5N1 avian influenza epidemic (Pohlmann et al. 2023), it was not possible to sample seabirds during 2023. All ticks were collected and stored in absolute ethanol until morphological identification. Morphological identification was done under a light microscope (Leica MS 5) according to published taxonomic keys (Estrada-Peña et al. 2017). Prior to DNA isolation, ticks were individually washed for 5 min in each 4% H_2_O_2_, 100% ethanol, and sterile ddH_2_O. Each tick was then bifurcated along the longitudinal and sagittal axes using a sterilized scalpel prior to sample digestion. Due to available resources, genomic DNA from all *I. ricinus* ticks collected in 2023 was isolated either using a modified salt extraction protocol based on (Aljanabi and Martinez 1997) or using the Magna Pure LC RNA/DNA Kit (Roche, Mannheim, Germany) according to the manufacturer’s instructions for all *Haemaphysalis* (*Ha.*) *punctata* samples. To avoid any potential biases due to extraction method in 2024, genomic DNA for all questing ticks was extracted using the DNeasy Blood & Tissue kit (Qiagen, Hilden, Germany) according to the manufacturer’s instructions. After isolation, DNA quantity and quality was checked using a NanoDrop One spectrophotometer (ThermoFisher, Waltham, MA, USA). During DNA extraction of the 2024 samples, nine samples were lost corresponding to three *I. ricinus* and six *Ha. punctata*, and could not be included in later analyses.

For 2024 samples morphologically identified as *Alectorobius* (*Al.*) *maritimus*, genomic DNA from two larvae was isolated using the Magna Pure LC RNA/DNA Kit (Roche, Mannheim, Germany) according to the manufacturer’s instructions. As morphological identification of this tick can be challenging (Dupraz et al. 2016), we amplified a fragment of the 16S rRNA using a previously described protocol and primers (Halos et al. 2004). These sequences were aligned using MUSCLE v3.8.425 (Edgar 2004a, b) as implemented in Aliview v1.28 (Larsson 2014) to GenBank references for soft tick species known to parasitize seabirds (Dietrich et al. 2010) among other sequences (Accession numbers: MK555333.1, L34305.1, KX258879.1, KX685689.1, KT781507.1, KX781698.1, KX130783.1, MK059461.1, KP861242.1, KU663027.1, KU551919.1, KX668415.1, KY454706.1, MG970562.1, KX130782.1, KX825972.1, KX825959.1, KX825970.1, KY825209.1, KY825215.1, LC818860.1, MH513307.1, KU757069.1). Phylogenetic reconstruction was performed in MrBayes v. 3.2.6 (Huelsenbeck and Ronquist 2001; Ronquist et al. 2012) with ploidy set to haploid and generalized time-reversible (GTR) (Tavaré 1986) substitution model with inverse gamma-distributed rate variation. Three independent runs were launched and ran for 5 million generations. Convergence was checked with Tracer v. 1.7.1 (Rambaut et al. 2018). Consensus trees were built using the *sumt* command from MrBayes using a respective burn-in of 25%. Convergence to a single topology in all three independent runs was checked manually in FigTree v. 1.4.4 (http://tree.bio.ed.ac.uk/software/figtree/). The tree was rooted on the branch separating the genera *Argas* and *Alectorobius*.

All tick samples were tested for the presence of *Borrelia* (*B.*) *burgdorferi* sensu lato (s.l.), *Rickettsia* (*R.*) spp., *Francisella* spp. and *Francisella-*like endosymbionts, *Babesia* (*Ba.*) spp., and *Anaplasma* (*A.*) *phagocytophilum* using molecular methods. For *Borrelia*, genomic tick DNA was tested using a qPCR targeting the 23S intergenic spacer following a standard procedure (Strube et al. 2010). Species identification of *Borrelia*-positive samples was done through amplification of the housekeeping gene *recG* using a previously described protocol (Margos et al. 2008). Genomic DNA isolated from a *B. bavariensis* culture (Strain: PBN) was included as a positive control. PCR products were sent to the Max Planck Institute for Evolutionary Biology and sequenced on an Applied Biosystems 3500 xL Genetic Analyzer. Sequences were manually checked for ambiguous sites in FinchTV Version 1.4.0 (Geospiza, Inc.; Seattle, WA, USA; http://www.geospiza.com) and then compared to the PubMLST *Borrelia* database. For *Rickettsia*, a pan-*Rickettsia* qPCR was used to amplify part of the *gltA* gene (Wölfel et al. 2008). *Rickettsia* species were determined through a *R. helvetica* specific qPCR targeting the 23S-5S intergenic spacer region (Chitimia-Dobler et al. 2018) or through amplification of the *ompB* gene and subsequent Sanger sequencing (Roux and Raoult 2000). For all *Rickettsia*-specific PCRs, DNA isolated from a cell culture of *R. helvetica* (Strain: AS 819) was included as a positive control. The *ompB* sequences were then aligned using MUSCLE v3.8.425 (Edgar 2004a, b) as implemented in Aliview v1.28 (Larsson 2014) to GenBank references of known *Rickettsia* species (accession numbers: MT090696.1, MT090687.1, MF002538.1, OP375094.1, HM446489.1, HM446488.1, OR168636.1, MW478137.1, MW248727.1, MW248724.1, MF002551.1, MZ020773.1, AF123719.1, MZ020771.1, KT835119.1, MF002557.1, OR734631.1, OR687054.1, ON721208.1, PQ197209.1, KR608787.1, LC379474.1, LC388802.1, LC388796.1, MF624879.1). *Rickettsia canadensis* was included as an outgroup to root the phylogeny. Phylogenetic reconstruction was performed as described above for the 16S rRNA sequences. Presence of *Francisella* spp. and *Francisella*-like endosymbionts was tested using a published method (Gehringer et al. 2013) using the LightMix® kit according to the manufacturer’s instructions including positive and negative controls delivered with the kit (TibMolBiol, Berlin, Germany). For *Babesia*, tick DNA samples were screened for *Babesia* spp. using a previously published PCR protocol targeting an 18S rRNA *Babesia* gene fragment (Casati et al. 2006) with modifications as previously described (Schorn et al. 2011) and subsequent commercial sequencing (Eurofins Genomics GmbH, Ebersberg, Germany) with obtained sequences of positive samples compared to GenBank sequences (https://www.ncbi.nlm.nih.gov/genbank/). DNA from a *Ba. microti* positive bank vole (*Clethrionomys glareolus*) was used as a positive control. Finally, the presence of *A. phagocytophilum* was assessed using a real-time PCR targeting a fragment of the *msp2* gene on an AB-7500 Real Time PCR System (Applied Biosystems, Darmstadt, Germany) (Obiegala et al. 2014). DNA isolated from an *A. phagocytophilum* culture was included as a positive control.

All statistical analyses were performed in R v4.4.2 (R Core Team 2024). For infection prevalence values, we estimated 95% confidence intervals (CI) using the Clopper-Pearson method (command: *exactci*) in the package PropCIs (Scherer 2018). Distributions of the two questing tick species identified in this study (*Ha. punctata*, *I. ricinus*) between years within species and among species over both years were tested with Fisher’s exact tests for count data using the *fisher.test* command (R Core Team 2024).

## Results

**Fig. 2:**
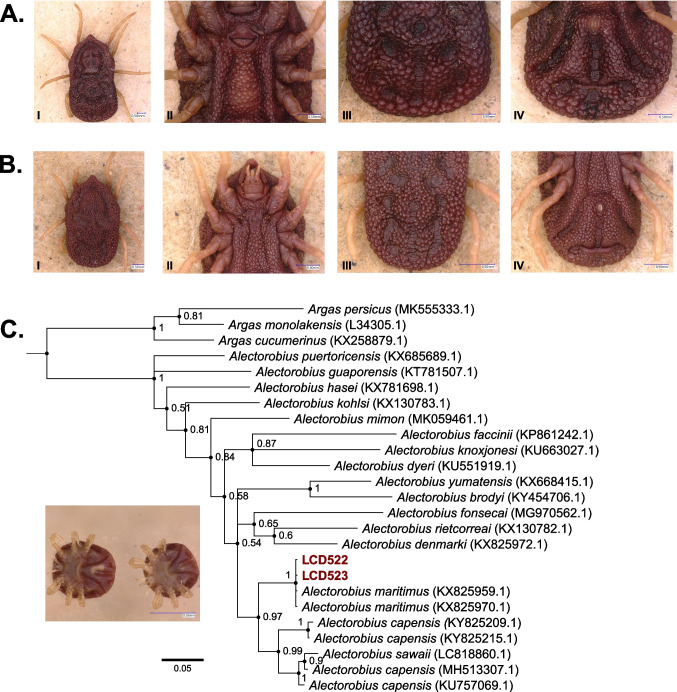
*Alectorobius maritimus* samples collected from common murre (*U. aalge*) chicks during the annual ringing effort of 2024. **A** Photos of the single adult tick sampled crawling on a bird: I, dorsal image; II, ventral image with focus on genital aperture; III, dorsal view with focus on mammillae; IV, ventral image with focus on anal groove. **B** Photos of the single nymphal tick sampled crawling on a bird: I, dorsal image; II, ventral image with focus on mouth parts; III, dorsal view with focus on mammillae; IV, ventral image with focus on anal groove. **C** Phylogenetic tree reconstructed based on the 16 s rRNA sequence amplified from two larvae (insert image) collected feeding on a single chick. The two samples (LCD522, LCD523) are shown in red. The scale bar is in substitutions per base pair and the numbers next to each node reports to the posterior probability of the node

In total, 267 ticks were collected in 2023–2024 and identified as *I. ricinus* (*n* = 132), *Ha. punctata* (*n* = 47), *I. uriae* (*n* = 3), and *Al. maritimus* (*n* = 85). Only *I. ricinus* and *Ha. punctata* were found questing on the island with adult and nymphal ticks of these species being found (Fig. 1B; Table 1). Besides a single nymph, all *I*. *ricinus* samples were adults (male and female) and were found in both sampling years in the NE-Gelände and Mittelland sampling locations (Fig. 1B; Table 1). The singular *I. ricinus* nymph was found in the Oberland sampling location in 2023, but with no further *I. ricinus* ticks being found at this site in 2024 (Table 1). *Haemaphysalis punctata* samples were predominantly collected at the Oberland collection site with very few being found in the NW-Gelände collection site in both years and the Südhafen collection site in 2024 (Fig. 1B; Table 1). Only *Ha. punctata* nymphs were found at the Oberland collection site (Fig. 1E) with adult females and nymphs (Fig. 1C, E) being found at the NW-Gelände site in both years and the Südhafen site in 2024 (Table 1). Additionally, a single adult male *Ha. punctata* (Fig. 1D) was collected from an employee of the Institute of Avian Research “Vogelwarte Helgoland” but was excluded from further analysis as the original questing location on the island could not be determined. From the three house pets sampled in 2024, 29 *I. ricinus* ticks were found, all of which were adults (male and female) (Fig. 1B; Table 1). For both *I. ricinus* and *Ha. punctata*, the number of ticks found at the three sites sampled in both years were not significantly different from each other within each species (Fisher’s exact test: *I. ricinus*, *p* = 0.305; *Ha. punctata*, *p* = 0.633). But overall, the distributions of *Ha. punctata* and *I. ricinus* in these plots did differ significantly from each other (Fisher’s exact test: *p* < 2.2 × 10^−16^). *Alectorobius maritimus* (*n* = 85) and *I. uriae* (*n* = 3) were both collected from five common murre chicks. For *Al. maritimus*, one adult (Fig. 2A), one nymph (Fig. 2B), and 83 larvae (Fig. 2C) were collected from four individual birds. All larvae shared the same morphology and phylogenetic reconstruction based on the 16S rRNA places the two sequenced larvae in a well-supported, monophyletic clade with the *Al. maritimus* reference sequences (Fig. 2C). For *I. uriae*, two nymphs and one larva were identified from a single common murre chick. These two tick species were not found infesting the same bird.

**Table 1:** Overview of collected ticks per year and sampling location on the North Sea island of Heligoland. Only the questing tick species (*Ha. punctata*, *I. ricinus*) are listed. In the table, tick life stages are listed as N = nymph, F = adult, female, and M = adult, male. The number next to the letter refers to the absolute number of ticks of this life stage of a specific species found at the sampling site in a given year

Year	Location	Tick species	Collected ticks
2023	Oberland	*Haemaphysalis punctata*	22N
		*Ixodes ricinus*	1N
	NW-Gelände	*Haemaphysalis punctata*	2F, 2N
		*Ixodes ricinus*	3F, 5 M
	Mittelland	*Haemaphysalis punctata*	-
		*Ixodes ricinus*	6F, 9 M
2024	Oberland	*Haemaphysalis punctata*	16N
		*Ixodes ricinus*	-
	NW-Gelände	*Haemaphysalis punctata*	1F
		*Ixodes ricinus*	16F, 13 M
	Mittelland	*Haemaphysalis punctata*	-
		*Ixodes ricinus*	29F, 19 M
	Südhafen	*Haemaphysalis punctata*	2F, 2N
		*Ixodes ricinus*	2F
	House pets	*Haemaphysalis punctata*	-
		*Ixodes ricinus*	20F, 9 M

Questing ticks (*I. ricinus*, *Ha. punctata*) were found positive for potentially pathogenic microorganism in both sampling years. *Ixodes ricinus* ticks tested positive for *B. burgdorferi* s.l. in both years corresponding to a prevalence of 0.39 (0.22–0.63; 95% CI) and 0.30 (0.21–0.39) in 2023 and 2024, respectively (Table 2). In comparison, *Borrelia*-positive *Ha. punctata* samples were only found in 2024 with a prevalence of 0.20 (0.04–0.48) (Table 2). *Haemaphysalis punctata* ticks though tested positive for *Rickettsia* spp. in both years corresponding to a prevalence of 0.154 (0.04–0.35) and 0.33 (0.12–0.62) in 2023 and 2024, respectively (Table 2). *Rickettsia*-positive *I. ricinus* samples were only found in 2024 corresponding to a prevalence of 0.11 (0.08–0.21) (Table 2). Very few *I. ricinus* tick tested positive for *A. phagocytophilum* in both sampling years, corresponding to a prevalence of 0.042 (0.00–0.21) and 0.01 (0.00–0.05) in 2023 and 2024, respectively (Table 2). Similarly, only two *I. ricinus* samples collected in 2024 tested positive for *Babesia* spp. corresponding to a prevalence of 0.02 (0.00–0.07) (Table 2). All ticks tested negative for *Francisella* spp. and *Francisella*-like endosymbionts. For a full overview of microorganism prevalence including 95% CIs and the absolute number of positive ticks for a given microorganism see Table 2.

**Table 2:** Prevalence of various potentially pathogenic microorganism tested for in questing ticks collected in 2023–2024 on the island of Heligoland. For each cell, the top number provides the overall prevalence in tested ticks, below this we report the 95% CI calculated using the Clopper-Pearson method (command: *exactci*) in the package PropCIs (Scherer 2018), and the final number provides the absolute number of ticks testing positive for the given microorganism. Any sample listed with a dash (-) refers to zero ticks testing positive for the microorganism. In the table, *n* = the total number of ticks collected, *Borrelia* = *Borrelia burgdorferi* s.l., *Rickettsia* = *Rickettsia* spp., *Babesia* = *Babesia* spp., *Anaplasma* = *A. phagocytophilum*, and *Francisella* = *Francisella* spp. and *Francisella*-like endosymbionts. During DNA isolation of the 2024 samples, nine samples were lost (*I. ricinus*, *n* = 3; *Ha. punctata*, *n* = 6) and were not able to be screened for tick-borne microorganisms

Tick species	Year	*n*	*Borrelia*	*Rickettsia*	*Babesia*	*Anaplasma*	*Francisella*
*Haemaphysalis punctata*	2023	26	-	0.15 (0.04–0.35) *n* = *4*	-	-	-
	2024	15	0.20 (0.04–0.48) *n* = *3*	0.33 (0.12–0.62) *n* = *5*	-	-	-
*Ixodes ricinus*	2023	24	0.39 (0.22–0.63) *n* = *10*	-	-	0.04 (0.00–0.21) *n* = *1*	-
	2024	105	0.30 (0.21–0.39) *n* = *31*	0.11 (0.08–0.21) *n* = *14*	0.02 (0.00–0.07) *n* = *2*	0.01 (0.00–0.05) *n* = *1*	-

**Fig. 3:**
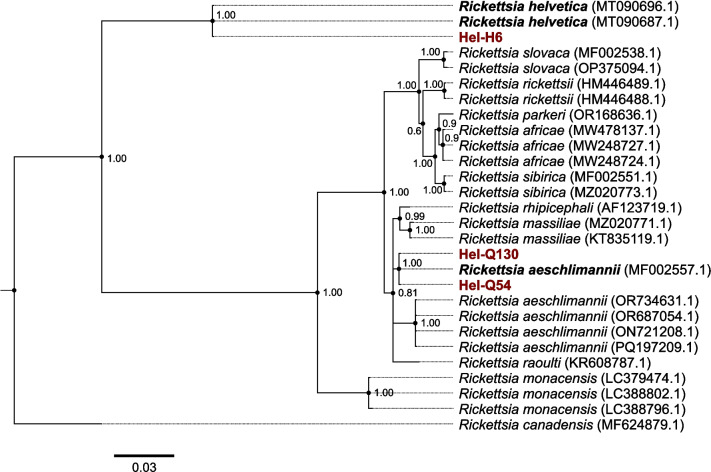
Phylogeny reconstructed based on the *ompB* sequence of *Rickettsia*. Samples from Heligoland (Hel-H6, Hel-Q130, Hel-Q54) are shown in red and the other samples are references downloaded from GenBank. The phylogeny was reconstructed using MrBayes v. 3.2.6 (Huelsenbeck and Ronquist 2001; Ronquist et al. 2012) with ploidy set to haploid and a GTR (Tavaré 1986) substitution model with inverse gamma-distributed rate variation. The tree was rooted on the branch leading to the outgroup *R. canadensis* included in the alignment. The scale bar is in substitutions per base pair and the numbers next to each node reports to the posterior probability of the node

Based on sequence comparison, the two *Babesia*-positive ticks were identified as *Ba. venatorum* and *Ba. capreoli*. Of the 44 *Borrelia*-positive ticks, we were not able to identify 15 of them to species level. Of the 29 samples for which we obtained a *recG* sequence, almost all showed ambiguous peaks in the trace files suggesting mixed infections (*n* = 22) with the closest allele type matches in the *Borrelia* PubMLST database being too isolates belonging to the species *B. valaisiana* (*n* = 13), *B. garinii* (*n* = 8), and *B. bavariensis* (*n* = 1). Of the remaining seven *Borrelia*-positive ticks, these were identified as *B. garinii* (*n* = 4), *B. valaisiana* (*n* = 2), and *B. afzelii* (*n* = 1). Only one of the three *Borrelia*-positive *Ha. punctata* could be determined and it showed a mixed infection with highest similarity to *B. garinii*. The four *Rickettsia*-positive ticks from 2023 could not be determined to species level as well as 11 of the ticks collected in 2024. Of the remaining *Rickettsia*-positive ticks, four were identified as *R. helvetica* in a species-specific qPCR. For three other samples the *ompB* gene was sequenced and phylogenetic reconstruction on this gene including GenBank references shows that one sample (Hel-H6) clusters with *R. helvetica* (Fig. 3) while the other two samples (Hel-Q54, Hel-Q130) form a well-supported, monophyletic clade with a reference sequence belonging to *R. aeschlimannii* (Accession Number: MF002557.1; Fig. 3). The *R. aeschlimannii* reference sequences included in our analysis are paraphyletic, but belong to a clade containing other spotted-fever *Rickettsia* species (e.g., *R. massiliae*, *R. rhipicephali*, *R. raoultii)* (Fig. 3).

## Discussion

We set out to determine the abundance of tick populations on the island of Heligoland located in the North Sea. During our collection period, we found four different tick species: *I. ricinus*, *Ha. punctata*, *Al. maritimus*, and *I. uriae*. From previous work, only the presence of *I. uriae* was already reported on the island (Rubel et al. 2021, 2023), while *I. ricinus* and *Ha. punctata* have only been observed feeding on migrant birds transiting through the island, but not actively questing (Walter et al. 1979). We supported for the first time the presence of a questing tick population positive for various, potential tick-borne pathogens (*Babesia* spp., *A. phagocytophilum*, *B. burgdorferi* s.l., *Rickettsia* spp.) showing that not only do ticks exist on the island, but also that they could pose a public health risk as vectors of various tick-borne pathogens.

From the ticks found, *I. ricinus* is a common tick with a wide distribution across all of Europe and parts of North Africa (Estrada-Peña et al. 2017) so it is not surprising that this species occurs on the island. In contrast, *Ha. punctata* is found more often in southern Europe around the Mediterranean Sea and has only been reported in three studies at five individual locations in Germany (Estrada-Peña et al. 2017; Rubel et al. 2023). Three of these locations were islands belonging to the Northern (Amrum) and Eastern Frisian Islands (Norderney, Juist) (Liebisch and Rahman 1976), which are all located on the German North Sea coast with also records of *Ha. punctata* on the Western Frisian Island of Texel in the Netherlands (Hofmeester et al. 2016; Medlock et al. 2018; Rubel et al. 2023) (Figure S1). This suggests that this tick species could be more common than previously thought in this region, with a potential affinity for island environments. *Haemaphysalis punctata* is known to have stable population in Southern England due to the milder climate caused by the Gulf Stream (Medlock et al. 2018), which may also extend to islands in the North Sea, like Heligoland, providing a suitable habitat for this tick. Both *I. ricinus* and *Ha. punctata* were found in both years in the same locations, but did differ significantly at which sites the two species were found suggesting some level of segregation of these two species on the island. In the case of *Ha. punctata*, both adult and immature stages of the ticks were found, which could suggest that this species is stable and is potentially able to reproduce using hosts present on the island. For *I. ricinus*, however, we found almost exclusively adult ticks on the island besides a singular nymph in 2023 (Table 2). Previous work has shown that *I. ricinus* larvae and nymphs can arrive to the island in high numbers on birds during their bi-annual migrations (Walter et al. 1979). This could mean that the adults found during the summers represent nymphs carried by migratory birds using Heligoland as a stopover site (Hüppop and Hüppop 2011) which have molted on the island. If this is the case, however, it will need to be checked in future research and monitoring efforts.

The other two tick species found, *I. uriae* and *Al. maritimus*, are known specialist tick species that utilize seabirds as their main host (Dietrich et al. 2010). The present work confirms previous reports of *I. uriae* on Heligoland (Rubel et al. 2021) and suggests that this tick is still present on the island. Our finding of *Al. maritimus* serves as the first finding of this tick species in Germany to the best of our knowledge (Rubel et al. 2021, 2023). As we observed all life-stages of this tick species (larvae, nymph, and adult) we assume that this tick is established on Heligoland and may use additional seabird hosts in addition to the common murre as the breeding colonies contain multiple bird species (Dierschke et al. 2011). *Alectorobius maritimus* is distributed along European coastlines and the Mediterranean Sea (Dietrich et al. 2010) with this finding potentially representing the furthest north that the species has been found and suggesting that this species may have a larger distribution than previously thought.

Within ticks collected actively questing on the island (*I. ricinus*, *Ha. punctata*), we found samples positive for multiple, potential human pathogens (*B. burgdorferi* s.l., *Rickettsia* spp., *Babesia* spp., *A. phagocytophilum*). The majority of *Borrelia*-positive *I. ricinus* showed mixed infections (i.e., presence of ambiguous peaks in Sanger trace file) with the only definitive samples being identified as *B. garinii*, *B. valaisiana*, and *B. afzelii*. Both, *B. garinii* and *B. afzelii* can act as a causative agent of Lyme borreliosis (LB) in humans (Stanek et al. 2011). *Borrelia valaisiana* has been detected through PCR in LB patients; however, if it is a causative agent of human LB remains debated (Diza et al. 2004; Margos et al. 2017). Of interest, though, is that the observed prevalence of *Borrelia*-positive ticks on Heligoland is higher than the European-wide average (Strnad et al. 2017). The increased prevalence on the island, especially in the case of *B. garinii* and *B. valaisiana*, could be a result of these ticks originating from migratory birds using the island as a stopover site. Thrush species, such as the Eurasian blackbird (*Turdus merula*), are the most common migratory bird observed on the island (Hüppop and Hüppop 2011) and can act as a reservoir host for both *B. garinii* and *B. valaisiana* (Kurtenbach et al. 2006; Norte et al. 2013), which could suggest why these species are found at such high prevalence. Further research is needed though to understand the host community structure on the island and correlate this to the increased *B. burgdorferi* s.l. prevalence. Both of the *Babesia* species identified in this study (*Ba. venatorum*, *Ba. capreoli*) can be pathogenic to humans or animals (Hildebrandt et al. 2021; Bajer et al. 2022). Even so, both of these species occur in our study at a very low prevalence, suggesting that disease risk could be reduced. This is also the case with *A. phagocytophilum* which can also cause disease in humans and animals (Dumler et al. 2005; Atif 2015). All three of these species are known to exist in Europe including Germany (Atif 2015; Bajer et al. 2022), but it remains unclear how they reached the island and if they are maintained, because important hosts, such as deer, are not present (Dierschke et al. 2011). We were not able to determine *Rickettsia* infection to species level in the four positive *Ha. punctata* ticks from 2023 and 11 *Rickettsia*-positive ticks in 2024. We could identify eight *Rickettsia* infections to species level in 2024 though. We found two species, *R. helvetica* and *R. aeschlimannii*, with only *I. ricinus* or *Ha. punctata* ticks being positive for either species, respectively. *Rickettsia helvetica* is a common microorganism found in *I. ricinus* and has been suggested to be pathogenic in some cases towards humans (Sprong et al. 2009; Gray et al. 2024). Hard ticks are known to carry also many *Rickettsia-*like endosymbionts, most of which are not pathogenic, which could also explain the unidentified *Rickettsia*-positive ticks observed here (Bonnet et al. 2017). *Rickettsia aeschlimannii* is a causative agent of spotted fever rickettsiosis in humans and is found in countries around the Mediterranean basin (Chisu et al. 2017; Seidi et al. 2024). In other studies, *Ha. punctata* ticks have been shown to carry *R. hoogstraalii* as well as other spotted-fever *Rickettsia*, but not *R. aeschlimannii* (Tijsse-Klasen et al. 2013; Chisu et al. 2017). Even though only two ticks were found positive for *R. aeschlimannii*, this finding does suggest a public health risk, and even though the prevalence of this and other pathogens remains low, future monitoring efforts are needed to determine the overall risk to public health.

It is not clear currently how both the questing tick species and potential pathogens found on the island arrived. More research will be needed to determine this fully, but both tick species have been found on migratory birds transiting through Heligoland (Walter et al. 1979), implementing migratory birds as a potential mode of introduction. This being said though, other work has suggested that tick movement can occur through tourism or even on pets and livestock (Hansford et al. 2016; Gillingham et al. 2020; Buczek and Buczek 2021). As Heligoland is often used as a tourist destination, this could also pose a viable mode of introduction (Dierschke et al. 2011). Regardless though, our results support a tick population on Heligoland and report the presence of *Al. maritimus* for the first time in Germany. We also confirmed that there are potential risks to public health through the presence of potentially pathogenic microorganisms. However, future monitoring schemes will be needed to track how this risk may develop over time.

## Supplementary Information

Below is the link to the electronic supplementary material.

Supplementary file1 (DOCX 51 KB)

## Data Availability

*Borrelia* recG sequences from 2023 have been uploaded to Genbank and can be found under the Accession Numbers: PP210028-PP210034. The remaining *Borrelia* and *Rickettsia* sequences produced in 2024 are in the process of being uploaded to Genbank but can be found along with individual tick collection data in the following Mendeley Data Repository: 10.17632/zrdfx36y4w.1.
