# Supplementary material for: Analysis of ticks (Acari: Ixodida) and associated microorganisms collected on the North Sea Island of Heligoland
Source: Parasitol Res. 2025 Mar 17;124(3):34. doi: 10.1007/s00436-025-08478-0 (PMC11914315; doi:10.1007/s00436-025-08478-0)
Supplement: Supplementary file 1 — Supplementary file1 (DOCX 51 KB) [file 436_2025_8478_MOESM1_ESM.docx]

**Analysis of questing hard ticks and associated microorganisms collected on the island of Heligoland**

**Parasitology Research**

Robert E. Rollins, Jochen Dierschke, Anna Obiegala, Heiner von Buttlar, Lidia Chitimia-Dobler, Miriam Liedvogel

- **Online Supplementary Material** —

**
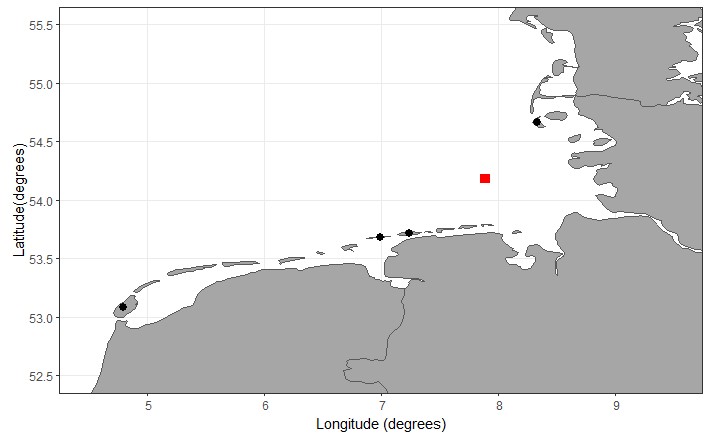
Figure S1.** Map showing geographic locations of published records (black circles) of *Haemaphysalis punctata* along the North Sea and the current finding of this tick species on the island of Heligoland (red square).
